# Advances in Pediatric Obsessive-Compulsive Disorder (OCD) Treatment: A Comprehensive Narrative Review

**DOI:** 10.7759/cureus.68225

**Published:** 2024-08-30

**Authors:** Ladan Khomami Zadeh, German Corso

**Affiliations:** 1 Medicine, Saint James School of Medicine, Valley, AIA; 2 Pediatric Psychiatry, Tropical Texas Behavioral Health, Harlingen, USA

**Keywords:** pharmacotherapy, ssris, cbt, treatment, pediatric, ocd, obsessive-compulsive disorder

## Abstract

Obsessive-compulsive disorder (OCD), characterized by persistent, intrusive thoughts (obsessions) and repetitive behaviors or mental acts (compulsions), can significantly impact a child's daily functioning, academic performance, and overall quality of life. As the prevalence of pediatric OCD continues to rise, there is a critical demand for evidence-based treatments that not only alleviate symptoms but also enhance the quality of life for affected children and adolescents. By identifying gaps in knowledge and suggesting directions for future research, this narrative review contributes to the ongoing discourse on pediatric OCD treatments. Ultimately, the synthesis of evidence aims to enhance our understanding and inform best practices in the compassionate and effective management of OCD in children and adolescents. The aim of this study is to provide a comprehensive overview of current trends and emerging strategies in the treatment of pediatric obsessive-compulsive disorder (OCD) and highlights the significance of tailoring treatment approaches to individual patient needs, considering factors such as symptom severity and treatment response. Concentrating on interventions supported by empirical evidence, the review delves into cognitive-behavioral therapy (CBT), pharmacotherapy, the synergistic effects of these modalities, and inventive therapeutic approaches, all while considering the distinctive developmental aspects pertinent to pediatric populations. We conducted this review by searching for titles in the PubMed database from 2013 to present. Our comprehensive literature review focused on advancements in treating pediatric OCD, using keywords like "Obsessive-compulsive disorder," "Pediatric," "treatment," "CBT," "SSRI," "Pharmacotherapy," and "combination therapy." While both pharmacotherapy and CBT show individual efficacy, the combination of these approaches appears to be more effective, especially for medication non-responders with no prior exposure to CBT, despite some mixed findings. These findings contribute significantly to the ongoing discussion on optimizing combined therapy strategies tailored to the complexities of pediatric OCD.

## Introduction and background

Obsessive-compulsive disorder (OCD) affects 1%-3% of children worldwide and has a profound impact on the quality of life for patients and families [1]. This condition is associated with affective and cognitive symptoms causing personal distress and reduced global functioning [2] and most often arises before adulthood with adolescence being a particularly vulnerable period [3]. Pediatric obsessive-compulsive disorder (POCD) is a chronic and impairing condition that often continues into adulthood. It is associated with more severe symptoms, a greater number of obsessive-compulsive symptoms, higher rates of comorbidity, a significantly higher prevalence of OCD among first-degree relatives, and generally poorer prognosis and response to treatment compared to adult-onset OCD. Additionally, having a family history of OCD has been linked to a six-fold decrease in the effectiveness of CBT monotherapy in treating POCD [4,5]. Despite the prevalence and severity of pediatric OCD, families encountering this challenge often face a significant "quality gap" in accessing evidence-based care, and even when services are reached, they are frequently delivered sub-optimally [6]. Pharmacotherapy, primarily employing serotonin reuptake inhibiting drugs (SRI), has been a cornerstone in pediatric OCD treatment over the past 30 years [7]. However, the efficacy of medications, including clomipramine, fluvoxamine, sertraline, and fluoxetine, shows a 30% to 40% reduction in symptoms, leaving a substantial proportion of patients with clinically significant residual symptoms [8]. Expert guidelines recommend cognitive-behavioral therapy (CBT) as the first-line treatment for mild to moderate pediatric OCD and a combination of CBT and selective serotonin reuptake inhibitors (SSRIs) for moderate to severe OCD [9]. CBT is also considered a first-line intervention for obsessive-compulsive disorder across the lifespan. Efficacy studies of CBT with exposure and response prevention suggest robust symptom reduction, often with sustained remission [10]. Many obsessive-compulsive and related disorders (OCDRDs) patients, however, fail to completely respond to first-line treatment with cognitive-behavioral therapy (CBT) and/or selective serotonin reuptake inhibitors (SSRIs) leaving practitioners with few additional treatment options [11]. Recognizing these limitations, current research explores novel avenues, including glutamate modulator agents such as N-acetyl cysteine (NAC) and deep brain stimulation (DBS), in the pursuit of more effective and comprehensive treatment strategies for pediatric OCD [12-14]. Glutamatergic agents are currently being studied for the treatment of OCD due to the glutamatergic pathway in the brain, related to OCD, and the role of the cortico-striato-thalamic circuit (CSTC) [13] and deep brain stimulation (DBS) has emerged as a treatment for severe cases of therapy-refractory obsessive-compulsive disorder, and promising results have been reported [14]. This article explores the diverse aspects of treating pediatric OCD, investigating present difficulties, established therapies, and the potential prospects of innovative interventions.

## Review

Methods

This narrative review was conducted by searching for titles in the PubMed database from the past decade (2013-2024). We comprehensively reviewed the literature on advances in the treatment of pediatric OCD, focusing on studies published in English. The search included keywords such as "Obsessive-Compulsive Disorder," "Pediatric," "Treatment," "CBT," "SSRI," "Pharmacotherapy," and "Combination Therapy." The following inclusion criteria were used to identify studies involving pediatric OCD, treatments, and intervention: psychotherapy including CBT, family-based cognitive-behavioral therapy (FB-CBT), and Internet cognitive-behavioral therapy (iCBT); and pharmacotherapy including SSRIs, N-Acetylcysteine, and Clomipramine and Combination therapy. Exclusion criteria were OCD with comorbid disorders, Pediatric Autoimmune Neuropsychiatric Disorders Associated with Streptococcal Infections (PANDAS) and Pediatric Acute-onset Neuropsychiatric Syndrome (PANS). For synthesis of information and management of search results, we manually reviewed a selection of articles to determine if all inclusion criteria were met. Results for each study that met our inclusion criteria were input into observation Table 1. We compared different therapeutic agents and their impacts on pediatric OCD.

**Table 1:** Summary of the Databases and Search Methods CY-BOCS: Children's Yale-Brown Obsessive Compulsive Scale, SRT: sertraline, OCD: obsessive-compulsive disorder, NAC: N-acetylcysteine, CBT: cognitive-behavioral therapy, ERP: exposure and response prevention, ICBT: Internet-delivered cognitive-behavioral therapy, FB-CBT: family-based cognitive-behavioral therapy, SSRI: selective serotonin reuptake inhibitor, DCS: D-cycloserine, N/A: not applicable.

Authors	Year	Intervention type	Age	Side effect	Type of study	Assessment tool for OCD outcome	Conclusion
Skarphedinsson et al. [15]	2015	Pharmacotherapy	7-17 years	Sertraline: Diarrhea and decreased appetite	Observational study	CY-BOCS	SRT treatment might be beneficial to a minority of patients who have consistently failed CBT.
Varigonda et al. [16]	2016	Pharmacotherapy	6-18 years	Clomipramine: weight gain, anticholinergic side effects, and arrhythmias	Meta analysis	NA	The greatest incremental treatment gains in pediatric OCD occur early in SSRI treatment.
Li et al. [17]	2020	Pharmacotherapy	8-17 years	NAC: Skin rash	Pilot study	CY-BOCS	There may be some initial improvement in OCD symptom severity with NAC treatment.
Wu et al. [18]	2016	Psychotherapy	5-18 years	N/A	Meta analysis	CY-BOCS	CBT is eﬃcacious in treating children's OCD.
Rosa-Alcázar et al. [19]	2015	Psychotherapy	<19 years	N/A	Meta analysis	CY-BOCS	The most promising treatments are those based on multicomponent programs comprising ERP, cognitive strategies, and relapse prevention.
Lenhard et al. [20]	2014	Psychotherapy	12-17 years	N/A	Open trial	CY-BOCS	ICBT could be eﬃcacious, acceptable, and cost-eﬀective for adolescents with OCD.
Babiano-Espinosa et al. [21]	2019	Psychotherapy	4-18 years	N/A	Systematic review	CY-BOCS	Evidence regarding acceptability, feasibility, and eﬃcacy of iCBT for pediatric OCD is limited, but results are promising.
Freeman et al. [22]	2014	Psychotherapy	5-8 years	N/A	Randomized controlled trial	CY-BOCS	A comprehensive FB-CBT program was superior to a relaxation program with a similar format in reducing OCD symptoms and functional impairment in young children (5-8 years of age) with OCD.
Mendez et al. [23]	2023	Combination therapy	Mean age: 12.7 ± 1.3 years and <18	N/A	Meta-analysis	CY-BOCS	Both SSRIs and SSRI+CBT produced early and sustained improvement.
Skarphedinsson et al. [24]	2016	Combination therapy	6-17 years	Clomipramine: harmful side effects SSRIs: 1. Gastrointestinal symptoms (e.g., loss of appetite, increased appetite, stomach pain, flatulence); 2. Suicidal thoughts and attempts; 3. Behavioral activation, especially in younger children. Second-generation antipsychotics: 1. Serious metabolic changes; 2. Weight gain; and 3. Sedation; 4. Mild to moderate behavioral activation with aripiprazole Clomipramine Combined with SSRIs: There is an increased risk of serotonin toxicity and cardiac arrhythmias.	Review	NA	The results did not indicate that combined treatment of CBT and SSRI is more eﬀective than CBT delivered by experts. However, combined treatment is more eﬀective than SSRI in SSRI non-responders.
Storch et al. [25]	2013	Combination therapy	7-17 years	Sertraline: decreased appetite and diarrhea.	Randomized controlled trial	CY-BOCS	Among youth with OCD, there was no evidence that sequentially provided sertraline with CBT diﬀered from those receiving placebo with CBT.
Cervin et al. [26]	2024	Combination therapy	6-17 years	SRIs: Gastrointestinal symptoms, weight gain, increased risk of suicidal thoughts.	Meta-analysis	NA	The combination of in-person CBT and SRIs may be most eﬃcacious, but few studies hinder firm conclusions.
Storch et al. [27]	2016	Combination therapy	7-17 years	No side effect reported	Randomized controlled trial	CY-BOCS	D-cycloserine augmentation of CBT did not confer additional benefit relative to placebo among youth with OCD.
Farrell et al. [28]	2013	Combination therapy	8-18 years	No side effect reported	Randomized controlled trial	CY-BOCS	DCS-augmented ERP produced significant improvements in OCD severity from posttreatment to one-month follow-up, relative to a placebo control condition, in severe and diﬃcult-to-treat pediatric OCD.

Results

**Figure 1:**
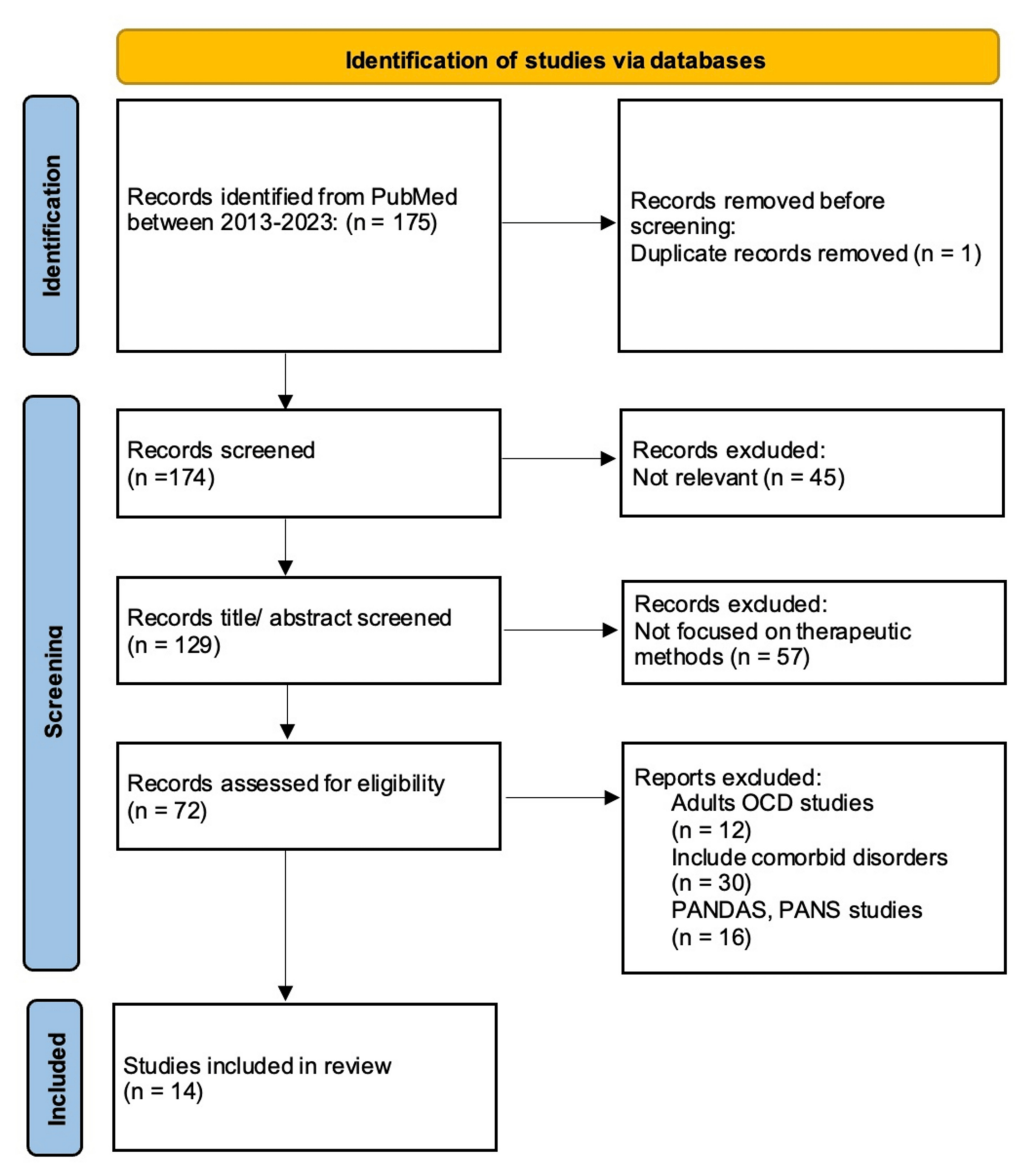
PRISMA Diagram PRISMA: Preferred Reporting Items for Systematic Reviews and Meta-Analyses, OCD: obsessive-compulsive disorder, PANS: Pediatric Acute-onset Neuropsychiatric Syndrome, PANDAS: Pediatric Autoimmune Neuropsychiatric Disorder Associated with Streptococcal Infections.

In this narrative study on pediatric OCD treatment, an initial pool of 175 articles was reviewed (see Figure 1).

After eliminating one duplicate, 45 articles were deemed irrelevant to the scope of our investigation. Subsequently, through a thorough examination of titles and abstracts, an additional 57 articles were excluded as they did not specifically address therapeutic methods for pediatric OCD. Furthermore, 12 articles focused on adult OCD treatment, leading to their removal from our analysis. Additionally, 30 articles discussing OCD with comorbid disorders and 16 articles centered on PANS and PANDAS were excluded. Following these steps, 14 articles remained for the final review, meeting the criteria for our in-depth analysis of therapeutic approaches in pediatric OCD treatment.

Discussion

Pharmacotherapy

The significance of pharmacotherapy in the treatment of pediatric obsessive-compulsive disorder (OCD) lies in its capacity to address symptoms and improve the overall well-being of affected individuals. While cognitive-behavioral therapy (CBT) remains a cornerstone in the management of pediatric OCD, pharmacotherapy offers valuable adjunctive or standalone options, particularly in cases where CBT alone may not provide optimal results. An investigation focused on assessing the impact of sertraline (SRT) in children and adolescents with obsessive-compulsive disorder (OCD) who had not responded to consecutive sessions of cognitive-behavioral therapy (CBT) [15]. This observational study, a part of the Nordic Long-Term OCD Treatment Study (NordLOTS), included 11 participants aged 7-17 with primary OCD. The participants underwent SRT treatment for a duration ranging from 72 to 300 days, with a mean of 164.2 days. The mean CY-BOCS score decreased from 21.5 to 17.5. Only three participants achieved an adequate clinical response (27.2%), and merely two participants achieved a reduction of more than 25% in the CY-BOCS total score, approaching 50%. The results indicated that, although the majority did not attain a sufficient clinical response to SRT, around one-third of the participants exhibited a clinical response. This suggests that SRT may offer benefits for a minority of patients who consistently failed to respond to CBT.

Similarly, the results from a meta-analysis of variations in efficacy among selective serotonin reuptake inhibitors (SSRIs) and between SSRIs and clomipramine in the treatment of pediatric obsessive-compulsive disorder (OCD) [16] highlighted that the optimal benefits from pharmacological treatment were observed early in the SSRI treatment course, aligning with findings observed in adults with OCD and in children/adults with major depression. This analysis incorporated nine trials, encompassing 801 children with OCD. The longitudinal data was best represented by a logarithmic model, indicating that the most significant benefits occurred early in the treatment for both clomipramine and SSRIs. Clomipramine demonstrated a greater measured benefit compared to placebo in comparison to SSRIs. Although data was limited, no evidence was found for a correlation between SSRI dosing and treatment effect. Both adults and children with OCD exhibited a similar degree and time course of response to SSRIs.

Moreover, a different avenue of investigation explored N-acetylcysteine (NAC), a drug that modulates glutamate, in a double-blind, placebo-controlled clinical trial involving children aged 8 to 17 with OCD [17]. NAC demonstrated a notable reduction in CY-BOCS total score compared to placebo, with effects starting to diverge from placebo by week 8. The mean CY-BOCS total score in the NAC group decreased from 21.4 ± 4.65 at baseline to 14.4 ± 5.55 at week 12. Conversely, in the placebo group, the mean CY-BOCS total score remained unchanged at 21.3 ± 4.65. Furthermore, within the NAC group, one out of five participants achieved over a 35% improvement in CY-BOCS total score, while none of the six patients in the placebo group reached this level of improvement. The study's findings suggested that NAC significantly reduced the severity of OCD symptoms compared to placebo, and the efficacy of NAC began to differentiate from placebo effects around week 8, indicating its potential promise in symptom alleviation. Collectively, these studies highlight the complexity of treating pediatric OCD and the potential benefits of pharmacotherapy, particularly in cases where individuals have not responded optimally to CBT alone. Sertraline, SSRIs, clomipramine, and NAC all present avenues worth exploring further for their potential in alleviating symptoms in pediatric OCD. However, larger-scale studies are needed to confirm and better understand the efficacy and tolerability of these pharmacological interventions in this population.

Psychotherapy

The efficacy of cognitive-behavioral therapy (CBT) for pediatric obsessive-compulsive disorder (OCD) has been extensively studied, with a focus on refining treatment protocols and exploring alternative modalities to enhance effectiveness and accessibility. Two meta-analyses have equivalently demonstrated the substantial role of cognitive-behavioral therapy (CBT) in the treatment of symptoms associated with obsessive-compulsive disorder (OCD) [18,19]. The efficacy of cognitive-behavioral therapy (CBT) on obsessive-compulsive disorder (OCD) was assessed by comparing post-treatment and pre-treatment scores on the Children's Yale-Brown Obsessive Compulsive Scale (CY-BOCS) [18]. Statistical evaluation of 13 studies utilized the weighted mean difference (WMD) and the results indicated a significant decrease in both CY-BOCS between pre- and post-CBT treatment in the overall database. These findings demonstrated a noteworthy reduction in scores after CBT treatment, signifying a substantial alleviation of OCD symptoms. In a complementary meta-analysis conducted in 2015, titled "Psychological treatment of obsessive-compulsive disorder in children and adolescents" [19], 46 published articles utilizing various forms of cognitive-behavioral therapy (CBT) were examined. The effect size for each group was calculated as the standardized pretest-posttest mean change, both for obsessive-compulsive symptoms and other outcome measures. The findings revealed substantial effect sizes for CBT in reducing obsessive-compulsive symptoms, and to a lesser extent, other outcome measures. The study underscored the effectiveness of CBT in significantly alleviating obsessive-compulsive symptoms. The exploration of treatment techniques highlighted the efficacy of multicomponent programs, incorporating exposure and response prevention, cognitive strategies, and relapse prevention as particularly promising.

Recognizing the significance of enhancing treatment accessibility, endeavors have been undertaken to extend the reach of cognitive-behavioral therapy (CBT). A notable strategy involves age appropriate therapist-guided Internet-delivered CBT (ICBT), exemplified by the "BiP OCD" platform [20]. In a feasibility and efficacy study, adolescents with OCD and their parents participated in a 12-week ICBT program. The treatment resulted in noteworthy improvements across all clinician, parent, and most self-administered outcome measures, demonstrating a substantial effect size on the Children's Yale-Brown Obsessive Compulsive Scale (CY-BOCS). Patients exhibited continued improvement during follow-up, with 71% classified as responders (≥35% decrease on the CY-BOCS) and 76% in remission (CY-BOCS score ≤12) at the six-month follow-up. These findings suggest that ICBT could represent a viable and cost-effective option for adolescents with OCD, addressing the need for alternative delivery methods, especially for those with limited access to traditional therapeutic settings. Following Preferred Reporting Items for Systematic Reviews and Meta-Analyses (PRISMA) guidelines, another systematic review aimed to evaluate the acceptability, feasibility, and efficacy of traditional cognitive-behavioral therapy (CBT) in conjunction with Internet-based CBT (iCBT) as a potential strategy to enhance treatment accessibility and outcomes in pediatric obsessive-compulsive disorder (OCD) [21]. Six identified studies within this research demonstrated promising results, showcasing a notable decrease in OCD severity and indicating high rates of feasibility and favorable acceptability of iCBT interventions. iCBT presents potential advantages by offering CBT in a format that reduces stigma and provides more widely available and accessible care. Moreover, tailoring the treatment to the expertise and cultural expression of young patients may enhance motivation and adherence to the program, potentially leading to more effective treatment and fewer dropouts. Exploring cost-effective and easily accessible autonomous treatment programs with minimal therapist contact is particularly intriguing within a stepped care model, allowing for differentiation between patients benefiting from low-cost treatments and those requiring therapist-delivered CBT.

Another innovative form of cognitive-behavioral therapy (CBT), specifically family-based CBT (FB-CBT), has shown similar outcomes in treating obsessive-compulsive disorder (OCD) among juvenile populations. FB-CBT focuses on equipping both the child and parent with the "tools" to understand, manage, and reduce OCD symptoms. The primary components of FB-CBT include (1) psychoeducation, (2) behavior management skills training for parents, (3) externalizing OCD and exposure plus response prevention (EX/RP) for children, and (4) family process components. The Pediatric Obsessive-Compulsive Disorder Treatment Study for Young Children (POTS Jr) conducted a randomized clinical trial to compare the efficacy of FB-CBT with a family-based relaxation treatment (FB-RT) control condition in children aged five to eight years. FB-RT focuses on using relaxation strategies to lower the child’s anxiety, with components including (1) psychoeducation, (2) affective education, and (3) relaxation training. [22]. Responder status was defined as achieving a score of 1 (very much improved) or 2 (much improved) on the Clinical Global Impression-Improvement scale, as well as a change in the continuous total score on the Children's Yale-Brown Obsessive Compulsive Scale as assessed by an independent evaluator. Family-based CBT showed superiority over FB-RT on both primary outcome measures, with 72% of children in the FB-CBT group rated as 1 or 2 on the Clinical Global Impression-Improvement scale at 14 weeks compared to 41% in the FB-RT group. These findings indicate that FB-CBT outperformed FB-RT in reducing OCD symptoms and functional impairment in this younger age group. Incorporating evidence from studies on CBT, ICBT, and FB-CBT, it is evident that various modalities show promise in addressing pediatric OCD. While traditional CBT remains a gold standard, innovative approaches such as ICBT demonstrate potential for reaching a broader audience, and tailored interventions like FB-CBT show efficacy even in younger children. Future research directions should continue to explore the optimal integration of these approaches, considering individual differences and treatment accessibility to maximize overall efficacy and improve outcomes for pediatric patients with OCD.

Combination Therapy

Combination therapy, involving the simultaneous use of multiple treatment modalities, holds significant importance in the treatment of pediatric obsessive-compulsive disorder (OCD). Multiple studies and meta-analyses have examined the efficacy of these modalities, shedding light on their relative effectiveness and the potential synergy of combined treatments. Studies exploring combination therapy with cognitive-behavioral therapy (CBT) and selective serotonin reuptake inhibitors (SSRIs) in the treatment of pediatric obsessive-compulsive disorder (OCD) present varied outcomes, with an overall indication of superior effectiveness compared to other treatments. A meta-analysis scrutinized the effects of CBT, SSRIs, and their combination in pediatric OCD [23]. The results suggested that both SSRIs and the combination of SSRI+CBT demonstrated early and sustained improvement over 12 weeks compared to placebo. Although the addition of CBT to SSRIs showed numerically greater improvement, the statistical significance of this difference was not established. Certain sample characteristics, such as more boys, younger patients, and greater baseline symptom severity, appeared associated with enhanced treatment response. Another review emphasized evidence-based treatments for pediatric OCD, underscoring the effectiveness of cognitive-behavioral therapy (CBT) and selective serotonin reuptake inhibitors (SSRIs) in patients new to treatment [24]. Robust evidence supports the efficacy of both CBT and SSRIs in treatment-naïve patients. Head-to-head trials reveal that CBT is notably more effective than SSRIs. Limited evidence for patients resistant to CBT or SSRIs suggests that these treatments remain effective for non-responders, and a combination of CBT and SSRIs emerges as the most effective approach for those resistant to SSRIs without prior exposure to CBT. 

Nevertheless, a contrasting outcome was reported in a different study that investigated the effectiveness of administering sertraline followed by cognitive-behavioral therapy (CBT) compared to CBT with a pill placebo over an 18-week period among children and adolescents diagnosed with obsessive-compulsive disorder (OCD) [25]. Forty-seven participants aged between 7 and 17 years were randomly assigned to one of three treatment arms: 1) standard-dose sertraline + CBT; 2) slowly titrated sertraline with at least eight weeks on the maximum tolerated daily dose + CBT; or 3) pill placebo + CBT. Outcome measures included the Children's Yale-Brown Obsessive-Compulsive Scale scores, as well as response and remission rates, Child Obsessive Compulsive Impact Scale-Parent/Child, Children's Depression Rating Scale-Revised, Multidimensional Anxiety Scale for Children, and Clinical-Global Impressions-Severity. The findings did not reveal any discernible differences between the group receiving sertraline followed by CBT and the group receiving placebo followed by CBT among youth diagnosed with OCD. 

Another recent study aimed to assess the effectiveness and acceptability of cognitive-behavioral therapy (CBT) and serotonin reuptake inhibitors (SRIs) for treating pediatric obsessive-compulsive disorder (OCD) through a network meta-analysis [26]. The analysis included 30 relevant randomized controlled trials (RCTs) and 35 comparisons involving 2,057 young individuals with OCD. The findings revealed that in-person CBT demonstrated significantly higher efficacy compared to Internet-based CBT, waitlist, relaxation training, and pill placebos. However, there were no significant differences between in-person CBT and CBT delivered via webcam/telephone, SRIs, or the combination of in-person CBT and SRIs. SRIs also were found to be more effective than pill placebos and waitlists. Therefore, the study suggests that in-person CBT and SRIs offer clear benefits compared to waitlist and pill placebos, advocating for their integration into the clinical management of pediatric OCD. In-person CBT, in particular, has a stronger evidence base. The combination of in-person CBT and SRIs may be the most effective, although limited data hinder firm conclusions.

The innovative concept of combining cognitive-behavioral therapy (CBT) with an alternative medication, D-cycloserine, examined in two studies, yielded conflicting results. A randomized clinical trial assessing the efficacy of weight-adjusted D-cycloserine augmentation alongside cognitive-behavioral therapy (CBT) for pediatric obsessive-compulsive disorder (OCD) [27] revealed that D-cycloserine did not yield additional benefits compared to placebo. Despite notable reductions in obsessive-compulsive symptoms, no significant disparities were observed between the D-cycloserine augmented CBT group and the placebo augmented CBT group, indicating that D-cycloserine augmentation may not augment treatment outcomes in pediatric OCD. Conversely, in a pilot trial targeting challenging-to-treat pediatric OCD cases [28], both exposure and response prevention (ERP) + D-cycloserine (DCS) and ERP + placebo (PBO) cohorts exhibited considerable post-treatment enhancements, with 94% responders. Notably, at the one-month follow-up, ERP + DCS demonstrated superior improvements in obsessional severity, diagnostic severity, and parent-rated OCD severity compared to ERP + PBO. However, these distinctions were not sustained at the three-month follow-up, suggesting transient benefits of DCS-augmented ERP in mitigating obsessional ideation, prompting further inquiry into severe pediatric OCD cases. Collectively, these studies and meta-analyses underscore the importance of considering individualized treatment approaches for pediatric OCD. While SSRIs and CBT individually exhibit efficacy, their combined use might not always yield significantly greater improvements in the short term. However, longer-term investigations are warranted to delineate the optimal timing and potential additive benefits of combining CBT with SSRIs, especially in cohorts with specific demographic or symptom severity profiles. Ultimately, these findings contribute to the ongoing dialogue on optimizing combination therapy strategies for pediatric OCD. Together, these studies emphasize the complexity of augmentative strategies in pediatric OCD treatment and the need for further exploration to refine and optimize interventions for this population.

Future Consideration

The future of pharmacotherapy and other treatments for pediatric OCD holds promise, with ongoing research exploring novel medications, targeted interventions, and advanced therapeutic modalities. Continued investigation into the efficacy and safety of emerging pharmacological agents, such as glutamatergic modulators and innovative psychopharmacological approaches, may provide additional options for tailoring treatment to individual needs. Moreover, the integration of neuroscience findings may lead to the development of more precise and personalized interventions, potentially utilizing neurostimulation techniques or neurofeedback to modulate neural circuits associated with OCD symptoms. The implications of this study suggest that expanding our understanding of these emerging treatments could enhance therapeutic strategies, offering more effective and individualized care. Collaborative efforts between researchers, clinicians, and technology experts are likely to pave the way for innovative, accessible, and effective treatments, ultimately improving the quality of care and outcomes for children and adolescents with OCD.

## Conclusions

The studies presented in this narrative review elaborate on the effect of pharmacotherapy, psychotherapy, and the combinatory effects of these methods on pediatric OCD. SSRIs, clomipramine, and glutamatergic modulators such as NAC and D-cycloserine all present avenues worth exploring further for their potential in alleviating symptoms in pediatric OCD. Incorporating evidence from studies on CBT, ICBT, and FB-CBT, it is evident that various modalities show promise in addressing pediatric OCD. Although traditional CBT remains a gold standard, innovative approaches such as ICBT demonstrate potential for reaching a broader audience, and tailored interventions like FB-CBT show efficacy even in younger children. Both pharmacotherapy and CBT demonstrate individual efficacy; however, despite certain mixed findings, the combination of these modalities emerges as more effective, particularly for medication non-responders without prior exposure to CBT. Ultimately, these insights contribute substantially to the ongoing discourse on optimizing combined therapy strategies tailored to the complexities of pediatric OCD.
